# Patient and provider perspectives on implementation barriers and facilitators of an integrated opioid treatment and HIV care intervention

**DOI:** 10.1186/s13722-019-0133-9

**Published:** 2019-01-28

**Authors:** Alexis Cooke, Haneefa Saleem, Saria Hassan, Dorothy Mushi, Jessie Mbwambo, Barrot Lambdin

**Affiliations:** 1 Department of Psychiatry, University of California, San Francisco, 3333 California Street, Suite 485, San Francisco, CA 94118 USA; 2 Johns Hopkins Bloomberg School of Public Health, Baltimore, MD USA; 3 Yale School of Medicine, New Haven, CT USA; 4 Department of Psychiatry, Muhimbili University of Health and Allied Sciences, Dar-es-Salaam, Tanzania; 5 RTI International, San Francisco, CA USA

**Keywords:** Care integration, Implementation, HIV, Substance use

## Abstract

**Background:**

In Dar es Salaam Tanzania, the first opioid treatment program (OTP) in Sub-Saharan Africa, had very high rates of enrollment of people who use drugs (PWUD) but low rates of antiretroviral therapy (ART) initiation among HIV-positive patients. The integrated methadone and anti-retroviral therapy (IMAT) intervention was developed to integrate HIV services into the OTP clinic. The objective of this paper is to better understand the contextual factors that influence the effectiveness of IMAT implementation using the consolidated framework for implementation research (CFIR).

**Methods:**

Semi-structured, in-depth interviews were conducted with 35 HIV-positive OTP patients and 8 OTP providers at the Muhimbili National Hospital OTP clinic 6-months after IMAT implementation. Providers were asked about their reactions to and opinions of the IMAT intervention including its implementation, their role in patient education, intervention procedures, and ART dispensing. Interviews with patients focused on their experiences with the IMAT intervention and adapting to the new protocol. Analysis of interview data was guided by the CFIR.

**Results:**

The CFIR constructs found to be driving forces behind facilitating or impeding IMAT implementation were: intervention characteristics (e.g. complexity, adaptability and evidence related to IMAT), outer setting (e.g. patient needs and resources), and inner setting (e.g. compatibility of IMAT and available resources for IMAT). The most significant barrier to implementation identified in interviews was availability of resources, including workforce limitations and lack of space given patient load. OTP providers and patients felt the design of the IMAT intervention allowed for adaptability to meet the needs of providers and patients.

**Conclusions:**

Understanding the contextual factors that influence implementation is critical to the success of interventions that seek to integrate HIV services into existing programs for key populations such as PWUD. Approximately 4 months after IMAT implementation, the OTP clinic adopted a ‘test-and-treat’ model for HIV-positive PWUD, which significantly impacted clinic workload as well as the care context. In this study we highlight the importance of intervention characteristics and resources, as key facilitators and barriers to implementation, that should be actively integrated into intervention protocols to increase implementation success. Similar interventions in other low-resource settings should address the ways intervention characteristics and contextual factors, such as adaptability, complexity and available resources impact implementation in specific care contexts.

## Introduction

Integrating HIV care and treatment services into opioid treatment programs (OTP) can improve linkages to HIV care and antiretroviral therapy (ART) and optimize HIV treatment benefits for people who use drugs (PWUD) [1]. However, there can be challenges specific to implementing care integration in setting with limited resources. Research indicates that adding or scaling up programs in these settings, can impact clinic efficiency and patient flow and without attention to structural barriers many patients might be lost to follow up [2, 3]. In integrating care services, particularly in setting with limited resources and among vulnerable populations, there is a need to figure out how to deliver and sustain these efforts in ways that are effective, timely and of high quality. Simple translation of interventions, from one care context to another, may not address issues of cultural appropriateness, resource limitations, existing health care structures, or political will.

PWUD shoulder a disproportionate burden of HIV in Tanzania [4]. HIV prevalence among PWUD living in Tanzania is estimated at 36% compared to 7% in the general population [5, 6]. In an effort to address the high rates of HIV among PWUD in Tanzania, the first publicly-funded OTP on the mainland of sub-Saharan Africa opened in February 2011 at Muhimbili National Hospital (MNH) in Dar es Salaam. The MNH OTP clinic offers methadone maintenance treatment, as well as psychosocial and behavioral therapies, as part of its medication-assisted treatment of opioid use disorder [7]. However, despite daily attendance at the MNH OTP clinic, less than half of treatment-eligible patients initiated ART within 3 months of being deemed eligible for treatment [8], due partly to delays in CD4 testing, suboptimal systems to monitor patients and link them to care, and siloed health care structures [9].

To address delays in ART initiation and improve HIV-related clinical outcomes among PWUD attending the MNH OTP clinic, we implemented the Integrated Methadone and Anti-retroviral Therapy (IMAT) intervention at the OTP clinic starting in 2015. At the launch of IMAT implementation, the IMAT intervention included four key components: (1) in-house point-of-care CD4 testing; (2) in-house HIV clinical management by methadone clinic providers trained in comprehensive HIV management, with referrals to the HIV clinic for developing needs; (3) ART delivery through the OTP clinic; and (4) an electronic information system to help providers monitor OTP patients along the continuum of HIV care. At the beginning of 2016, the Tanzanian Ministry of Health and Social Welfare adopted a ‘test and treat’ model for HIV among PWUD at the OTP clinic. Following this change more patients were eligible to initiate onto ART due to the elimination of reliance on a specific CD4 threshold to determine ART eligibility. Since this change occurred after IMAT it has effectively enabled providers at the OTP clinic to operationalize the first ‘test and treat’ model for HIV among PWUD in sub-Saharan Africa. In this paper, qualitative data were collected to examine provider and patient perspectives on the implementation of integrated methadone and HIV services at the Muhimbili OTP clinic.

## Methods

Semi-structured in-depth interviews were conducted with 35 HIV-positive OTP patients and 8 OTP providers and at the MNH OTP clinic 6-months after IMAT implementation. We interviewed providers at the OTP clinic who were involved with IMAT at the time of data collection, which included 3 nurses, 2 doctors, 1 pharmacist, 1 social worker and 1 administrative person. Providers were asked about their reaction to and opinions of the IMAT intervention including its implementation, their role in patient education, intervention procedures, and ART dispensing.

The 35 patients interviewed were purposively sampled based on sex and ART treatment status, and for those on ART, when they were linked to ART (Table 1). Interviews with patients focused on their experiences with the IMAT intervention and adapting to the new protocol. Patients were eligible for the study if they were HIV seropositive and enrolled in care at the OTP clinic at the time of data collection. Providers at the clinic determined if patients met eligibility requirements for in-depth interviews. Those who were eligible for study participation were asked if they were interested in participating during their private appointments, as to limit the risk of HIV status disclosure. Interviews were conducted in a private location and were audio-recorded, transcribed in Swahili, and then translated into English. This study received ethical approval from the Tanzania National Institute for Medical Research, the Muhimbili University of Health and Allied Sciences Institutional Review Board, and Ethical and Independent (E&I) Review Services in the United States.

**Table 1:** Characteristics of HIV-positive OST patients interviewed for the study (n = 35)

Patient characteristic	Men	Women	Total
HIV positive OTP clinic patients not on ART, post IMAT	1	2	3
HIV positive OTP clinic patients not linked to ART before IMAT, now on ART	23	9	32
Total	24	11	35

### Data analysis

Interview transcripts were entered into Dedoose (Version 7.0.23) for storage, organization, coding and analysis. The CFIR was used to guide data coding and analysis. Memos were used at each stage of data analysis to saturate analytic categories and facilitate analysis.

The CFIR framework includes five domains: intervention characteristics, outer setting, inner setting, characteristics of individuals involved and process [10]. In this study, we applied these domains to understand patient and provider perspectives on the context in which the intervention was delivered, rather than individual outcomes. Using available data, we focused on three out of five CFIR domains: intervention characteristics, outer setting, and inner setting. We did not apply the domain of process in this study because we were examining implementation at one site, and did not have data on quality and extent of planning, engagement of key stakeholders and did not conduct a multi-site comparison of health care delivery systems. Additionally, we did not apply the domain of characteristics of individuals involved because this domain is used to understand the behavior of the implementer or participant and its effect on implementation [11]. Of the CFIR constructs assessed, available data resulted in operationalization of eight implementation constructs: complexity, adaptability, relative advantage, evidence, patient needs and resources, available resources, and compatibility. These constructs and their operationalization, as defined by Damschroder [10] are presented in Table 2.

**Table 2:** Implementation constructs

CFIR domain	Implementation construct	Operationalized definition
Intervention characteristic	Complexity	The complexity (in terms of time, steps, or difficulty) of the intervention itself
	Adaptability	The ability, or lack thereof, to adapt the intervention to the specific clinic context
	Relative advantage	Improvements or worsening by the intervention, compared to currently existing programs, or having nothing in place of the intervention
	Evidence	Stakeholders’ perceptions of the quality and validity of evidence supporting the belief that the innovation will have desired outcomes
Outer setting	Patient needs and resources	The extent to which the intervention addressed or accounted for patient needs and limited resources
Inner setting	Available resources	The presence or absence of resources specific to the intervention
	Compatibility	The level of compatibility the intervention had with work processes and organizational values

Each interview was coded by the first author (AC), and these codes were used to develop case memos for each construct. Codes related to the construct of patient needs and resources included statements that discussed awareness, or lack of awareness about the needs and resources of those served by the intervention. Codes related to the construct of available resources included statements related to the presence or absence of resources specific to the intervention. Codes for compatibility included statements that discussed the level of compatibility the intervention had with work processes and organizational values. Statements were coded for the construct of complexity if they discussed the complexity of the intervention itself. Codes related to the construct of adaptability included statements regarding the ability, or lack thereof, to adapt the intervention to the specific clinic context. Codes for relative advantage included statements demonstrating that the intervention was better or worse than existing programs, or having nothing in place of the intervention. Lastly, codes for evidence included statements related to providers’ perceptions of the quality and validity of evidence supporting the belief that the innovation will have desired outcomes. Memos contained a summary of all relevant codes for the construct, a rationale for the code and the direct quotations related to the construct. From these memos we were able to assess the construct’s influence on IMAT implementation.

## Results

### Intervention characteristic

#### Complexity

In interviews with patients and providers at the OTP clinic, the complexity of the intervention was not endorsed as a barrier or facilitator to implementation. To some OTP providers, the complexity of the IMAT intervention was beneficial through its clearly defined protocol. However, other providers mentioned some procedures of the intervention that made it more difficult, at least, to initiate ART. For example, some lab services were not fully integrated into the OTP clinic procedures and initially registering patients to receive ARTs was difficult. The complexity of the intervention was not mentioned in interviews with patients. Patients did not mention that they felt burdened by the IMAT intervention protocol, nor did they mention that the intervention procedures were easy to follow.

#### Adaptability

OTP providers involved in the IMAT intervention felt that the ability of the intervention to be adapted to patient needs was a key facilitator of implementation. They spoke about the benefits of adaptability most frequently with regard to medication dispensing, which they felt was essential to improving patient adherence. Providers felt that adaptability of the intervention was critical in allowing modifications to the protocol that enabled it to be easily absorbed into pre-existing clinic procedures. This leveraged different provider roles while accommodating their time constraints and competing commitments. In interviews, providers’ perspectives regarding adaptability were discussed in terms of adaptations made to the IMAT protocol in terms of designating providers’ roles (e.g. who does blood draws and when) and prioritizing patients who needed to be seen by a clinician immediately. As one provider said:

So I can say that there are points where we have to diverge from the IMAT principles but it is explainable. There are a few of us so things have to be done in that way. The tests are run in the afternoon and the nurses prioritize patients, let’s say those who had lower CD4 counts were given priority when we were using the CD4 count category for initiating treatment.

In interviews patients also commented on the adaptability of IMAT implementation in regard to ART dispensing. Some IMAT patients were given the choice take their ART medication at home, rather than daily at the OTP clinic. Patients who were able to take ART at home appreciated the flexibility, which allowed them to take their medication with food, take them and be able to rest, or take them and be better able to deal with any side effects. This flexibility helps many patients, and also alleviates the number of patients a provider has to see every day in clinic. As one patient said:

I come for ART when they are finished. Some people were not taking their medications properly so it was concluded that they will be taking their medications here. Those who are doing well are given their medications to take at home. [My partner and I] are doing well and we are taking our medications at home. We ask them for a refill when they are finished.

This patient highlighted the importance of the intervention allowing patients to take the medications at home or through DOT based on their preference.

#### Relative advantage

Interviews with providers indicated that the benefits of the intervention compared to continuing care as usual, was seen as an implementation facilitator. Overall, providers felt the IMAT intervention resulted in a strong relative advantage to the alternative solution (non-integrated care). Providers felt that with IMAT, access to baseline laboratory studies, treatment and clinician referral was made much easier than prior to the intervention. Many of the providers also reported that IMAT procedures simplified and stream-lined their responsibilities and gave them the tools and resources needed to deliver care. As one provider commented:

It is not only one client, I remember so many clients. There were so many patients who were running [running away and leaving care] and so there was a delay in getting their blood samples for CD4 counts. This program has made it easy for us; we take the blood samples directly from the clients. We will do the other baseline investigations if the CD4 counts are found to be very low and we would call the doctor to start that specific client on therapy. The procedures used to take so long in the past and there were no special doctors for that group of clients. It is easier now because I can just call the doctor because there is a specific doctor for that and he can start the therapy [there is a specific doctor at the OTP clinic who initiates ART].

Patients also discussed the ways IMAT benefitted their care. In the 6 months since Patients receiving integrated care reported that they had already seen benefits to the care they received. From their perspectives, these improvements facilitated their adherence to the protocol and buy-into receiving HIV services, including ART dispensing, as part of their routine care at the OTP clinic. One patient described the ways integrated services improved their care at the OTP clinic:

I have an advantage on my health. My health has improved so much because I get all treatment at the same place. I am thankful for the providers because they are monitoring us closely. There are no disturbances when you get services at the same place.

#### Evidence

In interviews providers discussed improvements in care for HIV positive patients at the OTP clinic. Providers at the OTP clinic also discussed how the training they received about the IMAT intervention and integrating HIV care into the methadone clinic helped them understand the components of the intervention and supported implementation. While this training helped providers understand issues related to the low rates of ART initiation and barriers to ART integration that IMAT sought to address, early improvements to patients’ health served as evidence that IMAT could work in this specific care context. Providers felt confident in the intervention, as one provider explained:

I didn’t know if there would be any part of Africa that would provide the integrated services…I was aware of it after seeing it right here in our place. It opened up my mind and helped me understand better on how we can improve the MAT services and the ART services.

After participating in IMAT, patients also commented on improvements in their health. However, in interviews patients did not discuss their perceptions of the quality and validity of evidence against or in support of the IMAT intervention.

### Outer setting

#### Patient needs and resources

In interviews, providers endorsed feelings that the IMAT intervention was created with patient needs specifically in mind. Providers reported that the ability of the clinic to understand the needs and barriers of IMAT patients helped to facilitate intervention implementation. The IMAT intervention was created through a patient-engaged approach to address patient-level factors that impeded access to ART [7, 9]. Prior to the intervention, it was difficult for patients to initiate, obtain, and adhere to ART. Through integrating HIV care, OTP providers reported that they were better able to monitor patients’ health and identify factors that might impede treatment adherence. As one provider described IMAT:

I think the HIV services are better in the IMAT program. It has helped us to follow them up closely, we can tell if a client is not adhering to therapy and we have been able to establish the directly observed therapy. We observe them as they take [ART medication]. IMAT has enabled us to monitor them closely and start them on directly observed therapy if they are found with poor adherence.

In interviews with patients, they more frequently discussed how their unmet needs outside of the clinic might hinder their ability to engage in and adhere to treatment. Patients discussed feeling like these needs were not being addressed or met at the OTP clinic. Many of the patients at the clinic are unemployed and experience issues related to housing and transportation. For OTP patients enrolled onto IMAT lack of nutrition can severely hinder their ability to engage in all aspects of IMAT (e.g. reluctance to take ART without food) and adhere to treatment. As one patient described:

It is as I have told, how can I take methadone and the ARTs at the same time without taking food? I will fall down because both of the medications are strong. I am asking them to forgive me because you may hear that I have left their medications and have gone [patient left medications at the clinic and went home].

### Inner setting

#### Available clinic resources

In interviews providers discussed challenges related to the level of resources dedicated to intervention implementation. For example, the lack of technical resources such as a viral load machine hindered clinicians ability to monitor patients. OTP providers also discussed challenges related to workforce that limited their ability to carry out all intervention procedures. It is important to note, that these challenges might be a reflection of changes to workload following adoption of the ‘test and treat’ model. This unexpected shift increased the number of HIV positive OTP patients eligible for, and enrolled onto IMAT. While providers mentioned that there were features of the intervention that made providing care easier (e.g. centralizing many HIV-related services in the OTP clinic), they also felt that the intervention increased their workload (e.g. adding new responsibilities to the same number of existing staff). Some providers also specifically mentioned the lack of space coupled with large number of patients to manage, acted as a hindrance to implementation. As one provider described:

We, pharmacists, have no counselling rooms, we have no special room in which we can call clients and counsel them. We just end up feeling sad as we see patients deteriorating. We have no private room in which we can ask the patient why he is not taking his medications.

While there were some challenges around clinic resources reported by providers, patients enrolled in IMAT felt that the clinic had sufficient resources to provide HIV care. Many patients commented that IMAT made receiving HIV care and treatment easier and streamlined the ART initiation process. As one patient said:

I feel good because there are no circles in getting the medications. There are so many things to do in other hospitals. In here they just test you and start you on ARTs right away. Our doctor collects the medications for us. You just find your medications with him on your appointments.

#### Compatibility

Providers at the clinic felt that the intervention procedures were easily incorporated into the existing clinic workflow. The IMAT intervention leveraged the existing team structure in the OTP clinic, and strengthened provider collaboration as they sought to improve the care of HIV-positive patients. In interviews with providers, they spoke about how they were able to incorporate intervention procedures into existing workflows and systems at the clinic. As one provider said:

There is no gap because everybody has their own role to play. As a social worker, I know my roles at the clinic, the doctor knows his roles too. There are points in which I must consult the doctor or the nurse on duty. We can discuss about what to do about certain issues that may arise. We maintain confidentiality because it is very important. We can disclose some issues to the team members as we all work in the interest of the clients.

Patients at the OTP clinic felt that the structure and design of the IMAT intervention improved both HIV care receipt and monitoring. Patients also felt the IMAT intervention was designed in a way that addressed issues of confidentiality. From the perspective of patients, the IMAT intervention was integrated into the existing care system in a way that made it easy for patients to receive HIV care. As one patient described:

That is the first important thing that I want to say. It is very good that HIV services are integrated with methadone therapy. I just go to room number 11 for my medications and put them in my pocket. We have more confidentiality since the services have been integrated. The nurse goes to collect our medications for us. She comes here with the medications and we take them slowly. I am very happy with this system. It is a very good thing that HIV services have been integrated with methadone services.

## Discussion

In this paper we have used the CFIR to understand contextual barriers and facilitators related to implementation of the IMAT intervention. In-depth interviews with patients and providers 6-months post-implementation discussed how intervention characteristics, inner setting and outer setting factors influenced intervention implementation. Understanding the contextual factors that influence implementation is critical to the dissemination, scale-up and adaptation of the IMAT intervention to other settings.

Assessing context is important when evaluating interventions because it takes into account the fact that implementation outcomes neither exist nor arise in a vacuum. Context refers to the set of circumstances or unique factors that surround a particular implementation effort, and which must be accounted for in data interpretation [12]. Doing so allows us to assess whether intervention success or failure is related to problems with the protocol, or to the intervention occurring in a context without adequate resources, support, and the systems necessary for success. This is in alignment with implementation science literature that posits a clinic’s capacity to conduct an intervention does not necessarily reflect whether the intervention itself is effective or ineffective, but rather whether the intervention is implemented effectively and as intended in a particular context [13]. As applied to this study, we see differences between patients and providers, related to how context impacted intervention implementation. While the IMAT intervention was able to address patient needs in terms of ART initiation, more contextual factors such as food insecurity and transportation exist outside of the clinic’s purview, but still impact patient treatment and care. While these contextual factors may not impact the number of clients initiating ART it will certainly impact their medication adherence and therefore rates of viral load suppression which is a critical determinant of HIV related morbidity and mortality. Similar interventions might be able to facilitate implementation and create a more supportive environment through the expansion of services, allowing care to be located closer to where patients live (thus minimizing travel burden) and linking patients to relevant services, such as food assistance programs.

Facilitators of IMAT implementation included the CFIR domains of adaptability, relative advantage and evidence. These are all attributes of the intervention that were directly addressed by stakeholders (OTP patients, OTP providers, Local NGOs delivering HIV care services, HIV experts) at the time of intervention design [14]. This group identified ART initiation as a problem that could be addressed by integration of services. Furthermore, they highlighted the importance of designing an intervention that allowed for adaptability to meet the needs of providers (e.g. flexibility) and patients (e.g. confidentiality) as critical to the success of a complex intervention. The post-IMAT interviews confirmed that incorporating these stakeholders’ views provided for key facilitators for implementation. While not part of the intent of IMAT, interviews with patients and providers also suggest that integrating HIV care at the OTP clinic helped to improve retention as well. In looking to implement a similar integration program in other settings, it is important to incorporate stakeholder priorities in the intervention design to facilitate implementation.

The most significant barrier to implementation identified in interviews centered on contextual factors related to available resources. For some providers, lack of available resources (e.g. space) significantly hindered implementation and intervention delivery. While the shift to a ‘test and treat model’ was not explicitly discussed in interviews, it did result in increasing the number of OTP patients eligible for ART initiation. While providers discussed challenges related to available resources this was not a barrier endorsed in patient interviews. In interviews, IMAT patients felt they were able to easily receive services, which may indicate that IMAT patients are not acutely awareness of limitations in clinic resources or that providers were able to manage them without impacting their clinical care. In limited resource settings, prior service integration efforts in other health sectors have also shown the importance of policy-level efforts to improve the efficiency in resource utilization (providers and financial) of integration efforts [15]. These issues of available resources and compatibility will need to be addressed at the policy and service-delivery level, especially in context of a ‘test and treat’ model to ensure the sustainability and scalability of IMAT as well as other integration of service efforts.

In provider interviews we observed a discrepancy in comments about compatibility compared to comments made about available resources. While providers felt it was easy for them to incorporate intervention procedures into existing workflows and systems at the clinic, they also indicated that the level of resources (e.g. workforce, space and lab equipment) dedicated to implementation had an overall negative influence on implementation. The strain on resources may also been seen as a sign of successful implementation. Following the shift to a ‘test and treat model’, there was a sudden increase in the number of patients to manage. Had IMAT not been compatible with the OTP clinic, we might observe patients dropping out or leaving care thus not straining clinic resources in this way. This suggests that there may be tension between the compatibility of an intervention for a particular setting, and the necessary tools and resources needed to implement the intervention effectively. Having an adaptable intervention will allow for modifications to the intervention that will allow for increased compatibility for the available resources.

Though this study did not examine individual outcomes following IMAT implementation, we should note that OTP providers conducted over 100 CD4 tests within the first 3 months of IMAT implementation. Nearly 40 clients were seen by HIV care and treatment-trained clinicians for ART initiation at the MAT clinic. Following this the OTP clinic adopted a ‘test and treat’ model for HIV among PWUD at the OPT clinic, thus eliminating reliance on CD4 testing to determine ART eligibility and increasing workload demands [16].

There are limitations of this study. We were not able to the domain of process in this study due to lack of available data. Multi-site comparisons of intervention delivery may be a key component in understanding and assessing how implementation is carried out, as well as potential intervention barriers that occurred as a result of the implementation process. It is possible that the exclusion of this construct limits perspectives of implementation barriers and facilitators. We only interviewed patients currently enrolled and providers working at the MNH methadone clinic. Patients who have defaulted or who were not currently enrolled in care for other reasons may have very different perspectives around these issues. Thus, patient interview data may be biased in only collecting the experiences of those who have been successful in treatment. In addition, data for this study were collected in one setting, raising questions around generalizability.

## Conclusions

Despite these limitations, assessing determinants of implementation is critical to the replication of efforts in other settings. The CFIR constructs outlined in this paper were found to be driving forces behind facilitating or impeding IMAT implementation. Providers looking to integrate HIV care services into programs providing care for PWUDs in other settings in Sub-Saharan Africa should address intervention characteristics and critical inner and outer setting domains that will impact implementation. Continued use of CFIR in describing implementation of integration programs will allow for comparisons across studies as well as for improving the success of implementing evidence-based interventions.

## Abbreviations

PWUD: people who use drugs
OTP: opioid treatment program
MNH: Muhimbili National Hospital
ART: anti-retroviral therapy
IMAT: integrated methadone and anti-retroviral therapy
CFIR: consolidated framework for implementation research
CTC: care and treatment clinic
